# Positive covariation between current reproduction and subsequent performance in a raptor: Is the devil in the details?

**DOI:** 10.1002/ecy.70132

**Published:** 2025-06-02

**Authors:** Marlène Gamelon, Bertrand Scaar, Léo Dejeux, Sandrine Zahn, Josefa Bleu

**Affiliations:** ^1^ Laboratoire de Biométrie et Biologie Evolutive UMR 5558, CNRS Université Claude Bernard Lyon 1 Villeurbanne France; ^2^ Ligue pour la Protection des Oiseaux (LPO) Alsace Rosenwiller France; ^3^ Université de Strasbourg, CNRS, IPHC UMR 7178 Strasbourg France

**Keywords:** age, allocation to reproduction, *Athene noctua*, capture–recapture models, sex, trade‐offs

## Abstract

The theory about reproductive trade‐offs suggests that as reproduction is costly, individuals should trade current reproduction against future reproduction or survival, leading to within‐individual negative covariation between current reproduction and future performance. Despite clear predictions at the individual level, within‐individual negative covariations do not always translate into negative covariations at the population level: the devil may be in the details. For instance, if one sex only exhibits negative covariations between current reproduction at *t* and performance at *t* + 1, the covariation at the population level may be null or positive. Similarly, ignoring age effects may prevent the detection of negative covariations between current reproduction and subsequent performance at the population level. For a monogamous species with biparental care of young, a negative covariation between current and subsequent demographic performance is expected, similar for both sexes, but potentially stronger for the oldest senescent individuals. Here, we take advantage of a long‐term individual monitoring of male and female little owls (*Athene noctua*) and state‐of‐the‐art capture–mark–recapture models to assess covariation between vital rates between two consecutive years at the population level. When analyzing all individuals together, we found a positive covariation between reproduction in year *t* and survival or reproduction in year *t* + 1, regardless of sex, indicating that individuals with a high reproductive success in a given year tend to survive and reproduce better in the following year. This is an important finding because such positive covariations between demographic rates at the population level may overestimate the population growth rate. Looking more closely at individuals of known age within the population, we found evidence for age‐specific expression of reproductive trade‐offs. Population‐level covariation between current reproduction and subsequent demographic performance may thus mask more complex patterns of covariation between vital rates at finer scale.

## INTRODUCTION

According to the principle of allocation (Cody, 1966), individuals allocate their limited amount of resources to a function (maintenance, reproduction, growth) at the cost of other ones. At the individual level, this results in trade‐offs among fitness components, involving a negative covariation between traits (Williams, 1966). The most prominent life‐history trade‐off involves reproduction (Roff, 2002). The theory about reproductive trade‐offs suggests that as reproduction is costly, individuals should trade current reproduction versus reduced future survival (i.e., survival costs of reproduction) or fecundity (i.e., fecundity costs of reproduction). These costs can be paid either directly through immediate short‐term costs (between time *t* and time *t* + 1) (see Bleu et al., 2016 for a review in natural vertebrate populations), later in life through delayed long‐term costs inducing, for instance, accelerated senescence (Lemaître et al., 2015), or even transcend generations and be expressed on offspring performance (intergenerational costs) (Stearns, 1989, 1992).

Despite the clear theoretical prediction of negative covariations between current reproduction and subsequent performance at the individual level, a large number of empirical studies have provided evidence for null or even positive covariations at the population level (see Bleu et al., 2016; Hamel et al., 2010 for reviews). This may indicate the true absence of direct reproductive costs. But null or positive covariations can also hide more complex patterns within a population: the devil can be in the details. Indeed, individuals in a population may pay reproductive costs and thus show a negative within‐individual covariation between their current reproduction and subsequent performance. But if there is high among‐individual heterogeneity within the population, with, for example, some “bad quality” and “good quality” individuals, the covariation between current reproduction and subsequent performance observed at the population level may be null or positive, thus masking potential within‐individual trade‐offs (Hamel et al., 2010; van Noordwijk & de Jong, 1986). This means that positive covariation at the population level cannot always be interpreted as no within‐individual trade‐offs and thus as an absence of reproductive cost. However, when within‐individual trade‐offs are stronger than among‐individual variance in quality, the covariation at the population level can be negative. It is thus notoriously difficult to explore within‐individual trade‐offs from observational data in natural populations, and population‐level patterns of covariation between demographic rates should be interpreted with caution.

If one sex only exhibits negative covariations between current reproduction at *t* and performance at *t* + 1, assessing covariations without accounting for sex may wrongly indicate null or positive covariations at the population level. For example, depending on the strength of the sexual conflict over parental investment and the way this conflict is resolved, negative covariations in females can be associated with null or positive covariations in males (Lessells, 2012). Exploring covariations in both males and females in the same study is thus highly recommended. Up to now, most of the studies investigating life‐history trade‐offs involving reproduction in the literature have focused on females only because quantifying allocation to reproduction in males is more challenging than that in females. However, negative covariation between traits can also be observed in males (e.g., Bleu et al., 2016; Lemaître, Gaillard, et al., 2020; Scharf et al., 2013). Indeed, one can expect that a high level of paternal care should be costly, reducing subsequent survival or reproduction (Bennett & Owens, 2002; Trivers, 1972). For example, a meta‐analysis on experimental studies that manipulated brood size in birds indicates that males with high brood sizes exhibited reduced subsequent survival (Santos & Nakagawa, 2012). More generally, different roles of males and females in producing and raising the broods can induce sex‐specific reproductive costs.

Because allocation to reproduction can be age‐specific, accounting for age is also recommended while investigating covariations between current reproduction at *t* and performance at *t* + 1. For instance, senescence implying a progressive decline of demographic performance with age can lead to lower reproductive output at old ages (Lemaître, Ronget, et al., 2020; Nussey et al., 2013). Likewise, in primiparous individuals, reproduction can be lower than for experienced adults. Ignoring age effects may prevent the detection of negative covariations between current reproduction and subsequent performance at the population level (Cam et al., 2002). Moreover, negative covariations between reproduction and subsequent performance can occur for specific ages, not all, as shown in Asian timber elephants (*Elephas maximus*), where the cost of producing a young dramatically increases for old individuals (Robinson et al., 2012).

**FIGURE 1:**
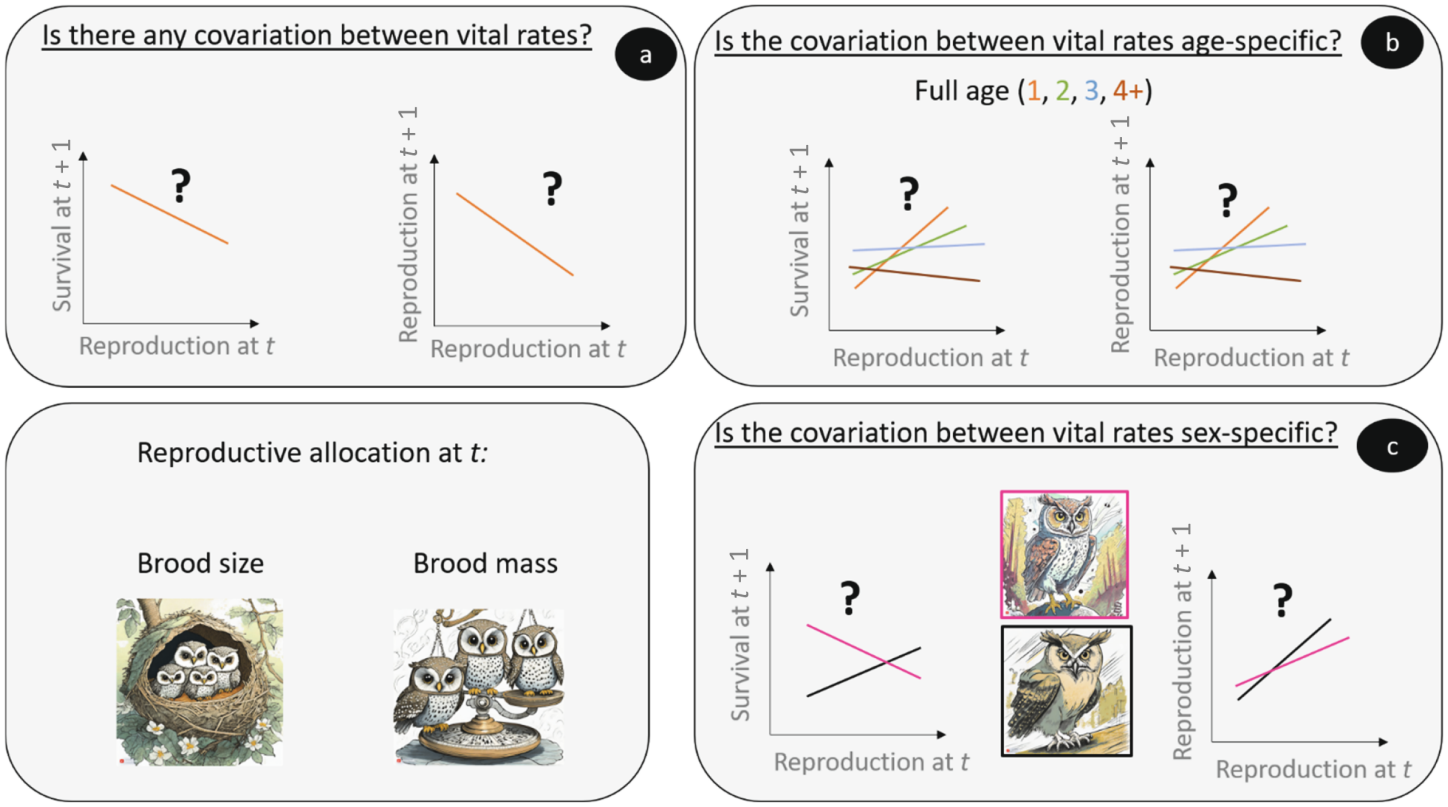
Schematic overview of the different analytical steps to explore covariations between reproduction at *t* and subsequent performance (reproduction, survival). Brood size and brood mass are used as measures of reproductive allocation at *t*. (a) Analysis performed on the whole dataset; (b) analysis performed on the subset of known age individuals; (c) analysis performed on the subset of individuals of known sex. Illustrations created by Marlène Gamelon using Adobe Firefly AI.

In this study, we assessed covariation between current and subsequent demographic performance in a raptor species, the little owl (*Athene noctua*). Taking advantage of the long‐term monitoring of free‐ranging individuals, we explored whether high allocation to reproduction at year *t* is associated with reduced survival and fecundity the following year while accounting for possible sex and age differences (see Figure 1 for a schematic overview of the approach). We used multistates and multievents capture–mark–recapture (CMR) models. States are discrete time‐varying individual covariates (e.g., breeder/non‐breeder) (Lebreton et al., 2009; Schwarz et al., 1993), whereas multi‐event models allow for uncertainty in state assignments (Gimenez et al., 2012; Pradel, 2005). Allocation to reproduction at time *t* can be measured in several ways. One simple way is obviously to assess whether an individual has reproduced or not. In that case, breeding status (non‐breeder vs. breeder or failed breeder vs. successful breeders) can be used as a proxy of reproductive allocation (Le Bohec et al., 2007; Sandercock et al., 2000; Vanderwerf, 2008). More fine‐tuned measurements of reproduction can be used. Here, we used brood size to quantify reproductive allocation at time *t*. However, two broods of similar sizes but different total brood masses represent different amount of energy allocated to reproduction. Therefore, we also used brood mass to measure reproductive allocation at time *t*. This species is monogamous and shows biparental care of the young; hence, we predict to observe similar negative covariation between current and subsequent performance in both sexes with possibly stronger negative covariations for older (senescent) birds.

## MATERIALS AND METHODS

### Model species and study site

The little owl is a small nocturnal raptor living in open or semi‐open areas, such as farmland or orchards (van Nieuwenhuyse et al., 2008). The little owl is territorial and breeds in cavity, including artificial nest boxes, once per season. This species is monogamous (no case of extrapair fertilization is reported in the literature) (Brouwer & Griffith, 2019; Müller et al., 2001; van Nieuwenhuyse et al., 2008) and characterized by biparental care (van Nieuwenhuyse et al., 2008). Little owls have a maximum longevity of 11 years in the wild in Europe (Fransson et al., 2017), and the average lifespan varies between 3 and 5 years (van Nieuwenhuyse et al., 2008).

The studied little owl population is located in the Alsatian territory (France). Since 2006, ringers and volunteers from the French League for the Protection of Birds (LPO) have installed and maintained more than 1500 nest boxes (Appendix S1: Figure S1). Females lay 2–6 eggs in April, hatching occurs ca. 1 month later.

### Data collection

The population has been surveyed following a national protocol from 2006 to 2021 (Hameau et al., 2015). Each year between February and July, nest boxes were visited, nestlings were ringed between 15 and 35 days of age, they were weighed with an electronic balance to the nearest 0.1 g, tarsus length was measured with a caliper to the nearest 0.1 mm, and the length of the third primary feather was measured with a ruler to the nearest mm. The number of nestlings in the nest was recorded (hereafter, brood size). We also collected 3–6 ventral coverts for molecular sexing of some of the nestlings (see below). Feathers were stored in ethanol 70% at ambient temperature during fieldwork and then at 4°C in the lab. The nestling dataset consists of 4672 owlets ringed (1449 broods, 592 missing data for tarsus length, 539 missing data for body mass, and 37 missing data for brood size). For each brood, we tried to capture the parents from February to July. To reduce disturbances, mothers with eggs or chicks younger than 5 days were not captured. Adults may be sexed thanks to the presence of the brood patch for females during incubation and rearing of the nestlings or using molecular sexing (see below). Adults were identified (if ringed as a chick) or ringed, and they were measured as described above for nestlings (tarsus length and body mass). The adult dataset consists of 1313 captures of birds (these raw data may include several captures of the same bird for a given year). In 2006 and 2007, there were only three captures of adults, whereas the number of captures ranged between 12 and 211 the other years (see Appendix S1: Table S1), and thus, we discarded the years 2006 and 2007 from the dataset. Adult individuals captured for the first time in 2021 were discarded from the analysis as they did not provide any information in terms of survival to the next year.

### Molecular sexing

Genomic DNA was extracted from feathers using an adapted protocol of the NucleoSpin Tissue kit (Macherey Nagel, Düren, Germany). Briefly, 0.5‐ to 1‐cm piece from one to three feathers per individual were cut in small pieces with a sterilized scissor. For samples containing unlysed quills after digestion, we centrifuged briefly, and we transferred the supernatant to another tube before proceeding with step 4 of the standard protocol.

Molecular sexing of nestlings was determined using the extracted DNA (following Griffiths et al., 1998). Briefly, the technique is based on the existence of two conserved CHD (chromo‐helicase‐DNA‐binding) genes that are located on the sex chromosomes. The CHD‐W gene is located on the W chromosome (only in females) and the CHD‐Z gene is located on the Z chromosome (both in males and females). The PCR amplification was carried out in a total volume of 20 μL. We used the following reagents: 0.3 units of Taq polymerase in the reaction buffer B (Euromedex), 1.75 mM MgCl_2_, 200 μM of each dNTP, and 100 μM of each primer (5′‐CTCCCAAGGATGAGRAAYTG‐3′ and 5′‐TCTGCATCGCTAAATCCTTT‐3′). Between 50 and 250 ng of genomic DNA was used as template.

### Capture–mark–recapture analyses

We used brood size and brood mass as measures of reproductive allocation.

#### Brood size analyses: Multistate model

For five captures of adult birds, information on brood size was missing, and thus, these data were discarded. The final dataset consisted of 607 adult individuals (125 non‐sexed individuals, 382 females, and 100 males) and 781 captures with a reproductive success from 663 different broods.

The median brood size was 3 (SD = 1.8). Thus, individuals with a brood size of 1, 2, or 3 were considered to have a low reproductive success and individuals with a higher brood size (4, 5, 6, or 7) to have a high reproductive success. The individuals with no associated reproductive success were either failed breeders or non‐breeders and were grouped together. Therefore, individuals can be in one of the four following states: alive with no reproductive success (“N”, state noted “1” in the CMR histories), alive with a low reproductive success (“L”, state noted “2”), alive with a high reproductive success (“H”, state noted “3”), and dead (“D”). For the states N, L, or H, individuals have a probability to be captured (*p*_*c*_) or not (1 − *p*_*c*_). When not captured, individuals are noted “0” in their history. For the state “D”, individuals are necessarily not captured (always noted “0”).

**FIGURE 2:**
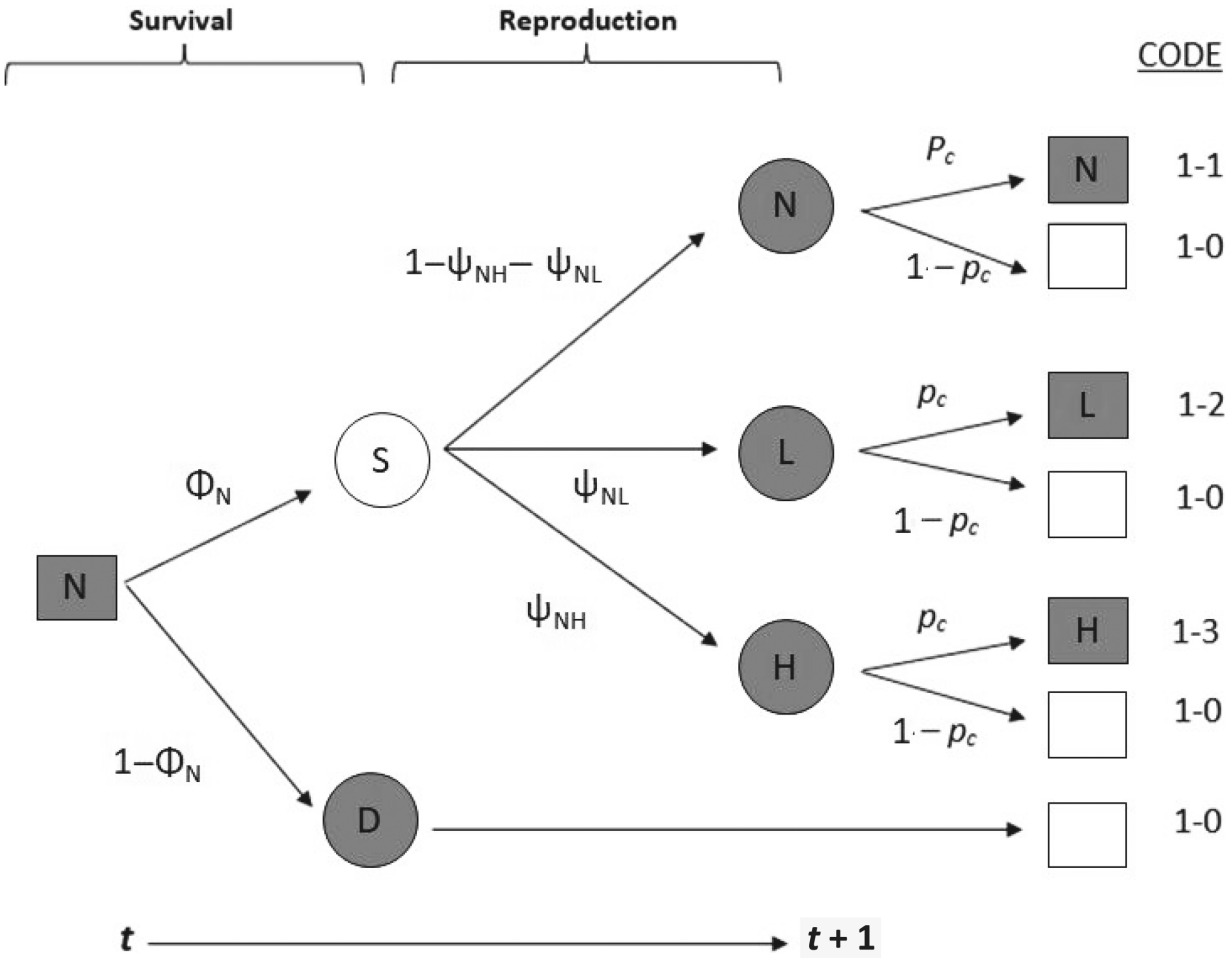
Fate diagram for individuals starting at year *t* with no reproductive success (departure state N) (brood size model). Individuals can survive (S) or die (D) until year *t* + 1. For surviving individuals, they can move toward the reproductive state “L” (low reproductive success), “H” (high reproductive success), or remain in the same state “N” (no reproductive success) at year *t* + 1. These states can be observed when the individuals are captured (with a probability *p*_*c*_) and are not observed when the individuals are not captured. States are figured with gray circles (D, N, L, H). The intermediate state S (surviving individuals) is figured with a white circle. Events are figured with squares. Fate diagrams with other departure states are shown in Appendix S1: Figures S2 and S3.

Individuals in a given reproductive state at year *t* (“N”, “L”, or “H”) can either survive with a probability Φ (Φ_N_, Φ_L_, or Φ_H_ depending on the reproductive state) or die (1 − Φ) until next year. Then, they have a probability to remain in the same reproductive state or to change to another state (“N”, “L”, or “H”) (ψ). Finally, they have a probability to be captured (*p*_*c*_), irrespective of the reproductive state (see fate diagrams in Figure 2, Appendix S1: Figures S2 and S3). We assumed recapture probabilities to be constant over time. This assumption allows reducing the number of parameters estimated by the model. We used a multistate CMR model to estimate survival probabilities Φ from breeding season *t* to breeding season *t* + 1, the transition probabilities ψ between reproductive states from *t* to *t* + 1 and capture probabilities *p*_*c*_. The two transition matrices, one for survival and one for reproductive states, are provided in Appendix S1: Figure S4. The event matrix allowing capture probabilities to be estimated is also provided in Appendix S1: Figure S4.

We defined a set of 12 models to test different biological hypotheses with regard to covariation between demographic performance (Table 1). Models estimating one survival parameter Φ between *t* and *t* + 1 (M1, M4, M7, M10) whatever the reproductive state at *t* assumed the same survival for all birds and thus null covariation between reproduction at *t* and the following survival. Models with two survival parameters, one for successful breeders Φ_SB_ (Φ_SB_ = Φ_L_ = Φ_H_) and another for others (Φ_N_) (M2, M5, M8, M11), assumed that successful breeders have different survival than other individuals. If Φ_SB_ < Φ_N_, it means that successful breeders at *t* have a lower survival between *t* and *t* + 1, indicating negative covariation. Models with three survival parameters Φ_N_, Φ_L_, Φ_H_ (M3, M6, M9, M12) assumed that individuals have different survival depending on their reproductive state at *t* (also called departure state, “N”, “L”, or “H”). If Φ_H_ < Φ_L_ < Φ_N_, this means that the higher the reproductive success at *t*, the lower the survival between *t* and *t* + 1, thus indicating negative covariation between reproduction at *t* and survival between *t* and *t* + 1.

**TABLE 1:** Models testing for covariation between reproduction at *t* and subsequent demographic performance in the little owl population, the associated number of parameters, and their biological meaning.

Model ID	No. parameters	Estimated survival and breeding probabilities	Biological meaning
M1	4	Φ/ψ	All individuals have the same survival and reproductive probabilities
M2	5	Φ_SB_ Φ_N_/ψ	Different survival probabilities between successful breeders and non‐successful breeders at *t* All individuals have the same reproductive probabilities
M3	6	Φ_N_ Φ_L_ Φ_H_/ψ	Different survival probabilities between the three states at *t* All individuals have the same reproductive probabilities
M4	5	Φ/ψ_SB_ ψ_N_	All individuals have the same survival probabilities Different breeding probabilities between successful breeders and non‐successful breeders at *t*
M5	6	Φ_SB_ Φ_N_/ψ_SB_ ψ_N_	Different survival and breeding probabilities between successful breeders and non‐successful breeders at *t*
M6	7	Φ_N_ Φ_L_ Φ_H_/ψ_SB_ ψ_N_	Different survival probabilities between the three states at *t* Different breeding probabilities between successful breeders and non‐successful breeders at *t*
M7	6	Φ/ψ_N_ ψ_L_ ψ_H_	All individuals have the same survival probabilities Different breeding probabilities between the three states at *t*
M8	7	Φ_SB_ Φ_N_/ψ_N_ ψ_L_ ψ_H_	Different survival probabilities between successful breeders and non‐successful breeders at *t* Different breeding probabilities between the three states at *t*
M9	8	Φ_N_ Φ_L_ Φ_H_/ψ_N_ ψ_L_ ψ_H_	Different survival and breeding probabilities between the three states at *t*
M10	9	Φ/ψ_NL_ ψ_NH_ ψ_LL_ ψ_LH_ ψ_HH_ ψ_HL_	All individuals have the same survival probabilities Different breeding probabilities between the three states (including states at *t* and at *t* + 1)
M11	10	Φ_SB_ Φ_N_/ψ_NL_ ψ_NH_ ψ_LL_ ψ_LH_ ψ_HH_ ψ_HL_	Different survival probabilities between successful breeders and non‐successful breeders at *t* Different breeding probabilities between the three states (including states at *t* and at *t* + 1)
M12	11	Φ_N_ Φ_L_ Φ_H_/ψ_NL_ ψ_NH_ ψ_LL_ ψ_LH_ ψ_HH_ ψ_HL_	Different survival probabilities between the three states at *t* Different breeding probabilities between the three states (including states at *t* and at *t* + 1)

*Note*: Survival probabilities Φ (and, respectively, breeding probabilities ψ) could be the same for all individuals (M1, M4, M7, M10 and, respectively, M1, M2, M3), could be different between successful breeders (SB) and non‐successful breeders (N) (M2, M5, M8, M11 and, respectively, M4, M5, M6), or could be different for the three reproductive states “L”, “H”, “N” (M3, M6, M9, M12 and, respectively, M7, M8, M9). Finally, all breeding probabilities could be different (i.e., depending on departure [at *t*] and arrival [at *t* + 1] reproductive states, M10, M11, M12).

Similarly, models with one reproductive parameter ψ at *t* + 1 (M1, M2, M3) whatever the reproductive state at *t* indicate null covariation between reproduction at *t* and *t* + 1. Models with two reproductive parameters at *t* + 1 (M4, M5, M6), one for successful breeders at *t* (ψ_SB_, where ψ_LL_ = ψ_LH_ = ψ_HL_ = ψ_HH_ and ψ_SB_ = ψ_LL_ + ψ_LH_ = ψ_HL_ + ψ_HH_) and another for non‐successful breeders at *t* (ψ_N_ where ψ_NL_ = ψ_NH_ and ψ_N_ = ψ_NL_ + ψ_NH_), indicate negative covariation between reproduction at *t* and *t* + 1 if ψ_SB_ < ψ_N_. Similarly, models with three reproductive parameters ψ_N_, ψ_L_ (where ψ_LL_ = ψ_LH_ and ψ_L_ = ψ_LL_ + ψ_LH_), and ψ_H_ (where ψ_HL_ = ψ_HH_ and ψ_H_ = ψ_HL_ + ψ_HH_) indicate different probabilities of breeding successfully at *t* + 1 depending on the reproductive state (“N”, “L”, or “H”) at *t* (M7, M8, M9). The last three models M10, M11, and M12 with six reproductive parameters (ψ_NL_, ψ_NH_, ψ_LL_, ψ_LH_, ψ_HH_, ψ_HL_) indicate different probabilities of breeding with a high or a low success at *t* + 1 depending on the reproductive state at *t*, with evidence for negative covariation if ψ_HH_ or ψ_HL_ < ψ_LL_ or ψ_LH_ < ψ_NL_ or ψ_NH_. We compared the 12 models based on their Akaike information criterion (AIC) value. The most parsimonious model is the model with the lowest AIC. If several models had comparable AIC (difference of AIC < 2), we retained the model with fewer parameters.

We performed the analysis on the entire dataset (step 1, Figure 1). Moreover, to account for potential age differences in vital rates (survival, reproduction) as well as in covariation between demographic performance, we analyzed a reduced dataset with 396 individuals of exact known age (i.e., marked as nestlings) (step 2, Figure 1). Age was included as an interactive effect on both survival and reproduction, and 4 age classes were used: age 1 (151 individuals), age 2 (99 individuals), age 3 (51 individuals), age 4 and older (95 individuals). Including “age” as an interactive effect in the models allows some ages to show negative covariations, while other ages show positive covariations between demographic performance, which is important to test our prediction of stronger negative covariation for older individuals. By contrast, additive effects of age would have constrained the ages to have the same covariance between vital rates (e.g., all ages would have showed positive covariations, or all ages would have showed negative covariations), preventing any detection of age‐specific reproductive trade‐offs. Finally, to account for potential sex differences, we analyzed a reduced dataset including individuals of known sex (382 females and 100 males), that is, individuals sexed at capture or by molecular sexing (step 3, Figure 1). As done for age, sex was included as an interactive effect on both survival and reproduction. We considered sex‐dependent recapture probabilities.

The analyses were performed with E‐Surge software (version 2.2.3) (Choquet, Rouan, et al., 2009). We tested the goodness‐of‐fit (GOF) (Pradel et al., 2003) using U‐Care software (Choquet, Lebreton, et al., 2009). None of the global tests were rejected for the whole dataset, the subset of known age, or the subset of known sex individuals (see Appendix S1: Table S2). Therefore, following the decision tree provided by Gimenez, Lebreton, et al. (2018), we concluded that the GOF test indicated no lack of fit.

#### Brood mass analysis: Multievent model

The dataset consisted of 611 adult individuals (127 non‐sexed individuals, 383 females, and 101 males) and 786 captures with a reproductive success from 668 different broods. Offspring are captured and weighed at different ages toward the end of their growing phase, and thus, their mass is not directly comparable. To compute brood mass, we standardized offspring mass by using the Scale Mass Index (SMI) as a measure of body condition (see Peig & Green, 2009, 2010). The SMI is a metric that adjusts the mass of individuals as if they all had the same size. We adjusted the mass to the median tarsus length (35.1 mm) and used a robust estimator of the coefficient of the standardized major axis regression line (library smart 3.4‐8 in R). Brood mass was then calculated as the sum of SMI of each nestling in a nest. The median brood mass was 424.16 g (SD = 158.7). Individuals producing a brood mass lighter than 424.16 g were thus considered to have a low reproductive success and individuals with a heavier brood mass to have a high reproductive success. As before, individuals with no associated reproductive success were either failed breeders or non‐breeders and were grouped together. Individuals can be in four different states (alive and high reproductive success “H”, alive and low reproductive success “L”, alive and no reproductive success “N”, dead “D”). Because for some of the broods, the mass was not recorded, we defined five different events at capture: captured and assigned with a high reproductive success (noted “3”), captured and assigned with a low reproductive success (noted “2”), captured and assigned with no reproductive success (noted “1”), not captured (noted “0”), and a new event: captured and reproductive success is low or high but is not assigned (brood mass is unknown, noted “4”). We used a multievent CMR model, which accounts for uncertainty in state assignment (Pradel, 2005) to estimate survival probabilities Φ from breeding season *t* to breeding season *t* + 1, the transition probabilities ψ between reproductive states from *t* to *t* + 1, capture probabilities *p*_*c*_, and the probability of being captured with a known brood mass γ_B_. This latter parameter γ_B_ allows dealing with mass uncertainty of some broods (Appendix S1: Figures S5–S7). The matrices are provided in Appendix S1: Figure S8. The model selection procedure was similar to that for brood size. As for brood size, we performed the analysis on the entire dataset (step 1, Figure 1), on a reduced dataset with 397 individuals of exactly known age (step 2, Figure 1), and on a reduced dataset including individuals with known sex (383 females and 101 males) (step 3, Figure 1). All the datasets are provided in Bleu (2025).

## RESULTS

### Covariation between brood size at *t* and subsequent demographic performance

**FIGURE 3:**
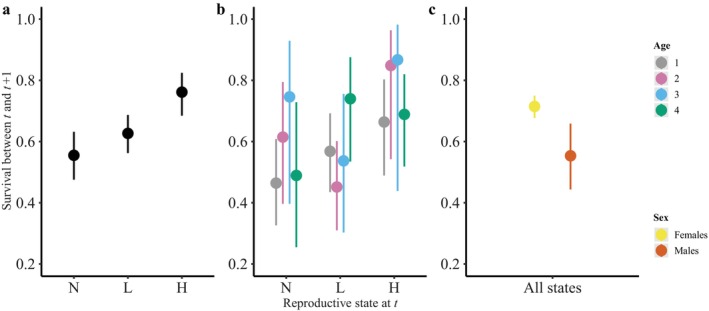
Survival probability between year *t* and *t* + 1 according to the reproductive state (brood size) at year *t* when the analysis is performed on the whole dataset (a), on individuals of known age only (b), and on individuals of known sex only (c). Displayed are the estimates provided by the best model retained (see Table 2). Reproductive state at year *t* corresponds to no (N), low (L), or high (H) reproductive success.

When the analyses were performed on the whole dataset, the best model indicated different survival probabilities among individuals that had no, low, or high reproductive success in terms of brood size (model M6, Table 2). In particular, individuals with no reproductive success at year *t* had the lowest survival between *t* and *t* + 1 (mean [95% CI], 0.56 [0.48, 0.63]), individuals with a low brood size exhibited a higher survival probability between *t* and *t* + 1 (0.63 [0.56, 0.69]), whereas individuals with a high brood size had the highest survival probability (0.76 [0.68, 0.82]) (Figure 3a). This indicates that the higher the reproductive success at *t*, the higher the survival probability between *t* and *t* + 1. This demonstrates a positive covariation between reproduction at *t* and survival from *t* to *t* + 1. The recapture probability was estimated to be 0.51 [0.47, 0.56]. The same analysis replicated on the dataset of known age individuals (M6_Φ(age)ψ(age), Table 2) revealed age‐specific covariation between reproduction at *t* and survival from *t* to *t* + 1 (Figure 3b). More precisely, while individuals of age 1 showed a positive covariation between reproduction at *t* and survival from *t* to *t* + 1 (Φ_N,age1_ = 0.46 [0.33–0.61], Φ_L,age1_ = 0.57 [0.43–0.69], Φ_H,age1_ = 0.66 [0.49–0.80]), this pattern was less clear for ages 2 and 3, for which individuals with a low brood size exhibited the lowest survival probability between *t* and *t* + 1 (Φ_L,age2_ = 0.45 [0.31, 0.60], Φ_L,age3_ = 0.54 [0.30, 0.76]). However, as for age 1, individuals of age 2 and 3 with a high brood size had the highest survival probability (Φ_H,age2_ = 0.85 [0.54, 0.96], Φ_H,age3_ = 0.87 [0.44, 0.98]) and individuals with no reproductive success had a lower survival (Φ_N,age2_ = 0.61 [0.40, 0.79], Φ_N,age3_ = 0.75 [0.40, 0.93]). For age 4, again, individuals with no reproductive success had the lowest survival from *t* to *t* + 1 (Φ_N,age4_ = 0.49 [0.25, 0.73]), while breeders that previously produced low or high brood size exhibited higher survival (Φ_L,age4_ = 0.74 [0.53, 0.88], Φ_H,age4_ = 0.69 [0.52, 0.82]). For the subset of individuals of known sex, the best model simply indicated different annual survival probabilities for females (0.71 [0.68, 0.75]) and males (0.55 [0.44, 0.66]) (model M4_Φ(sex)ψ(sex), Table 2, Figure 3c) whatever their previous reproductive status. The recapture probability was estimated to be 0.55 [0.50, 0.60] for females and 0.43 [0.29, 0.58] for males.

**TABLE 2:** Model selection for the effect of brood size at year *t* on parents' survival probability between *t* and *t* + 1 and the probability of successful reproduction at *t* + 1.

Dataset “all”		Dataset “age”		Dataset “sex”	
Model	AIC	Model	AIC	Model	AIC
M1	4234.57	M1_Φ(age)ψ(age)	2662.88	M1_Φ(sex)ψ(sex)	3625.70
M2	4230.00	M2_Φ(age)ψ(age)	2665.79	M2_Φ(sex)ψ(sex)	3629.39
M3	4225.61	M3_Φ(age)ψ(age)	2660.60	M3_Φ(age)ψ(sex)	3628.01
M4	4217.91	M4_Φ(age)ψ(age)	2656.07	M4_Φ(sex)ψ(sex)	**3617.46**
M5	4212.73	M5_Φ(age)ψ(age)	2659.49	M5_Φ(sex)ψ(sex)	3621.22
M6	**4208.30**	M6_Φ(age)ψ(age)	**2654.18**	M6_Φ(sex)ψ(sex)	3619.82
M7	4219.59	M7_Φ(age)ψ(age)	2657.81	M7_Φ(sex)ψ(sex)	3621.24
M8	4214.34	M8_Φ(age)ψ(age)	2660.78	M8_Φ(sex)ψ(sex)	3625.01
M9	4209.95	M9_Φ(age)ψ(age)	2655.44	M9_Φ(sex)ψ(sex)	3623.60
M10	4217.86	M10_Φ(age)ψ(age)	2663.93	M10_Φ(sex)ψ(sex)	3626.04
M11	4212.67	M11_Φ(age)ψ(age)	2666.43	M11_Φ(sex)ψ(sex)	3629.87
M12	4208.48	M12_Φ(age)ψ(age)	2661.02	M12_Φ(sex)ψ(sex)	3628.74

*Note*: Models are tested on the whole dataset including all individuals (all), on the dataset including individuals of known age only (age, with an age effect on survival probabilities Φ, breeding probabilities ψ, or both), and on the dataset including individuals for which the sex has been determined (sex, with a sex effect on survival probabilities Φ, breeding probabilities ψ, or both). See Table 1 for a full description of the 12 models (M1 to M12). Displayed are the AIC of each model tested. The most parsimonious models retained are in bold.

Abbreviation: Akaike information criterion.

**FIGURE 4:**
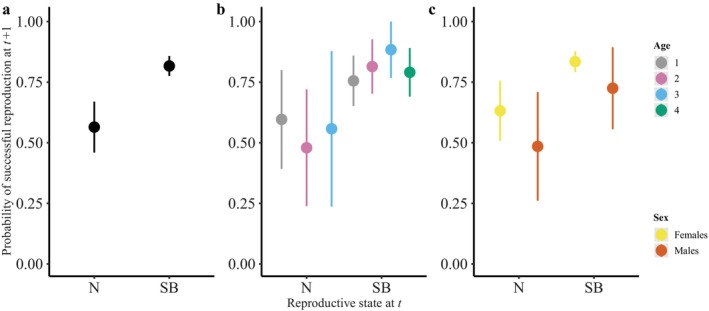
Probability of successful reproduction at year *t* + 1 according to the reproductive state (brood size) at year *t* when the analysis is performed on the whole dataset (a), on individuals of known age only (b), and on individuals of known sex only (c). Displayed are the estimates provided by the best model retained (see Table 2). Reproductive state at year *t* corresponds to non‐successful breeders (N) or successful breeders (SB).

In terms of covariation between reproduction at *t* and *t* + 1, when the analyses were performed on the whole dataset, the best model indicated that successful breeders at *t* had a higher probability of being successful breeders the following year (*t* + 1) (0.82 [0.78, 0.86]) than non‐successful breeders at *t* (0.56 [0.46, 0.67], model M6, Table 2, Figure 4a). This result suggests a positive covariation between reproduction at *t* and reproduction at *t* + 1. We found the exact same pattern for the analysis performed on individuals of known age (M6_Φ(age)ψ(age), Table 2, Figure 4b), for ages 1, 2, and 3. For the oldest age class (age 4 and older), the probability of being successful breeders at *t* + 1 for individuals with no reproductive success at *t* was not estimated by the model due to lack of power. For the subset of individuals of known sex, there was a positive covariation between reproduction at *t* and reproduction at *t* + 1 for both sexes (M4_Φ(sex)ψ(sex), Table 2, Figure 4c).

### Covariation between brood mass at *t* and subsequent demographic performance

When we explored the effect of brood mass produced at year *t* on parents' survival probability between *t* and *t* + 1 as well as on their probability of successful reproduction at year *t* + 1, we again found evidence for positive covariation. Indeed, the best model retained for the whole dataset (model M6, Table 3) indicated that individuals with no reproductive success at year *t* had the lowest survival between *t* and *t* + 1 (0.56 [0.48, 0.63]) and the lowest probability of being successful breeders at *t* + 1 (0.56 [0.46, 0.67]), whereas individuals with a high brood mass at *t* had the highest survival probability between year *t* and *t* + 1 (0.74 [0.66, 0.80]) and individuals that reproduced successfully at *t* (both low and high success) had the highest probability of being successful breeders at *t* + 1 (0.82 [0.78, 0.86]) (Appendix S1: Figures S9a and S10a). Thus, there is evidence for positive covariations between current reproduction and subsequent performance. The recapture probability was estimated to be 0.49 [0.44, 0.54], and the probability of being captured with a known reproductive state was estimated to be 0.88 [0.86, 0.90]. The same analysis replicated on the subset of individuals of known age (M4_Φ(age)ψ(age), Table 3) indicated age‐specific survival probabilities between *t* and *t* + 1, independent of previous reproductive states. As observed for brood size, we found a positive covariation between the brood mass produced at *t* and the probability of being successful breeders at *t* + 1 for ages 1, 2, and 3 (Appendix S1: Figures S9b and S10b). For the oldest age class (age 4 and older), the probability of being successful breeders at *t* + 1 for individuals with no reproductive success at *t* was not estimated by the model due to lack of power. For the subset of individuals of known sex, males and females had different survival probabilities (Appendix S1: Figures S9c and S10c), independent of their reproductive status at *t* (model M4_Φ(sex)ψ(sex), Table 3). As observed for previous analyses, there was a positive covariation between reproduction at *t* and *t* + 1 for both sexes.

**TABLE 3:** Model selection for the effect of brood mass at year *t* on parents' survival probability between *t* and *t* + 1 and probability of successful reproduction at *t* + 1.

Dataset “all”		Dataset “age”		Dataset “sex”	
Model	AIC	Model	AIC	Model	AIC
M1	4695.19	M1_Φ(age)ψ(age)	2938.25	M1_Φ(sex)ψ(sex)	4018.25
M2	4690.89	M2_Φ(age)ψ(age)	2941.60	M2_Φ(sex)ψ(sex)	4021.88
M3	4689.75	M3_Φ(age)ψ(age)	2945.89	M3_Φ(age)ψ(sex)	4019.31
M4	4678.39	M4_Φ(age)ψ(age)	**2931.58**	M4_Φ(sex)ψ(sex)	**4009.91**
M5	4673.50	M5_Φ(age)ψ(age)	2935.40	M5_Φ(sex)ψ(sex)	4013.60
M6	**4672.36**	M6_Φ(age)ψ(age)	2939.71	M6_Φ(sex)ψ(sex)	4011.03
M7	4680.38	M7_Φ(age)ψ(age)	2937.84	M7_Φ(sex)ψ(sex)	4013.49
M8	4675.51	M8_Φ(age)ψ(age)	2941.50	M8_Φ(sex)ψ(sex)	4017.14
M9	4674.37	M9_Φ(age)ψ(age)	2945.63	M9_Φ(sex)ψ(sex)	4014.68
M10	4676.80	M10_Φ(age)ψ(age)	2939.36	M10_Φ(sex)ψ(sex)	4014.21
M11	4672.08	M11_Φ(age)ψ(age)	2942.61	M11_Φ(sex)ψ(sex)	4018.02
M12	4671.01	M12_Φ(age)ψ(age)	2946.97	M12_Φ(sex)ψ(sex)	4016.07

*Note*: Models are tested on the whole dataset including all individuals (all), on the dataset including individuals of known age only (age, with an age effect on survival probabilities Φ, breeding probabilities ψ, or both), and on the dataset including individuals for which the sex has been determined (sex, with a sex effect on survival probabilities Φ, breeding probabilities ψ, or both). Displayed are the AIC of each model tested. See Table 1 for a full description of the 12 models (M1–M12). The most parsimonious models retained are in bold.

Abbreviation: Akaike information criterion.

## DISCUSSION

Using state‐of‐the‐art CMR models and a long‐term individual‐based monitoring, we assessed whether wild little owls exhibit negative covariation between current reproduction and subsequent performance. We expected negative covariations between reproduction at *t* and demographic performance at *t* + 1 for both sexes, especially for old birds. Direct costs of reproduction are the most prominent and well‐studied life‐history trade‐offs (Roff, 2002), but they are rarely explored for both sexes because assessing allocation to reproduction in wild males can be challenging (Archer et al., 2022; Bleu et al., 2016; Festa‐Bianchet, 2012). Here, using two measures of the amount of energy allocated to reproduction, namely, brood size and brood mass, for the whole dataset, we found a positive covariation between reproduction at time *t* and survival or fecundity at time *t* + 1, indicating that individuals with a high reproductive success a given year tend to better survive and better reproduce the following year, whatever their sex. This is a different pattern than the one described in another little owl population, where costs of reproduction were observed on survival during the breeding season. There was no effect of reproduction on survival to the following year except in low‐quality habitat (Michel et al., 2022).

Positive covariations among demographic rates are not an exception. This has been recently confirmed in a comparative study involving 15 bird and mammalian species, showing that positive covariations among demographic rates (e.g., survival, reproduction) are ubiquitous at the population level (Fay et al., 2022). Similarly, in a comparative study involving 40 plant species, Jongejans et al. (2010) found that positive covariances between reproduction and survival rates predominate. Going back to the seminal work from Tuljapurkar (1982) on the approximation of the long‐term stochastic growth rate, temporal variance in vital rates has a negative effect on the population growth rate. This negative effect of temporal variance on the population growth rate is enhanced if there is a positive correlation between vital rates (Hilde et al., 2020). Therefore, positive covariations at the population level are expected to decrease the long‐run population growth rate. This can have strong implications in wildlife conservation because ignoring positive covariations, as done in most studies (Earl, 2019), may overestimate the population growth rate (Earl, 2019; Fay et al., 2022). Their inclusion in population models thus seems crucial to provide accurate estimates of the population growth rate.

The positive covariations between current reproduction and subsequent performance we found for the analyses performed on the whole dataset can be explained by several factors/hypotheses not mutually exclusive. First, as expressed by van Noordwijk and de Jong (1986), when individual heterogeneity in resource acquisition is higher than individual heterogeneity in resource allocation, individuals that are able to acquire a large amount of resources may reproduce without apparent costs (good quality individuals) contrary to individuals in poorer conditions (Cam et al., 2002; Festa‐Bianchet et al., 2019; Gimenez, Cam, et al., 2018). This may explain the positive covariation between current reproduction and subsequent reproduction and survival observed in the studied population: some birds are always better at acquiring, allocating resources, and reproducing and surviving than others. Second, good conditions, such as favorable years or rich habitats where forage is more abundant, can result in the absence of trade‐off between current reproduction and future survival and reproduction (Michel et al., 2022; Rughetti et al., 2015; Townsend & Anderson, 2007).

Interestingly, our approach goes one step further by looking more closely at the intrapopulation level. Indeed, our modeling approach allowed us to compare demographic rates at year *t* + 1 for individuals with contrasting reproductive allocation at year *t* while accounting for sex and age effects. By looking more specifically at age‐specific trade‐off expression, we found some more complex patterns. For instance, while the positive covariation between brood size at *t* and survival the following year holds for age 1, individuals of age 2 and 3 with a low reproductive success at *t* tend to exhibit lower subsequent survival probability than individuals with no reproductive success at *t*. However, individuals with high reproductive success at *t* tend to exhibit the highest subsequent survival probability (Figure 3b). These results highlight that beyond the broad picture at the population level, some more complex patterns of covariations can exist.

In the monogamous little owl with biparental care, we expected negative covariation between current and future demographic performance for both males and females. We found evidence for positive covariation that was not sex‐specific. The absence of a sex effect is in accordance with a previous study on this species (Michel et al., 2022). While we used brood size and mass as a measure of allocation to reproduction, different reproductive stages may be associated with different costs. In birds, the relevant reproductive stages are mate acquisition, defense of the territory, nest building, egg production, incubation, and chick rearing. For example, it has been shown in female great tits (*Parus major*) that costs of incubation are higher than costs of egg production (Visser & Lessells, 2001). Interestingly, if the investment of the males and females differs between the reproductive stages, then we can expect sex‐specific reproductive costs even in monogamous species as observed in the European Storm‐Petrels (*Hydrobates pelagicus*): investment early in reproduction is different between males (territory defense) and females (egg production) and only females pay reproductive costs of first reproduction (Sanz‐Aguilar et al., 2012).

To conclude, in this study, we found that in a monogamous species with biparental care, positive covariations occur between reproduction at *t* and demographic rates at *t* + 1, even after accounting for sex effects. However, our results demonstrate that complex covariations between reproduction at *t* and subsequent demographic performances at *t* + 1 exist when accounting for age effects. Future studies could aim to better understand this pattern by studying the traits of the individuals or their territories that always succeed better than the others. This would allow to refine the concept of individual quality in this species. Controlling the analyses for “individual quality” could be helpful to assess within‐individual trade‐offs. Several methodological approaches can be used, such as adding individual ID as a random factor (van de Pol & Wright, 2009) or using a good proxy of individual quality (e.g., body mass) as a covariate. Doing so with CMR data in multistate/multievent models with per definition a detection probability lower than 1 remains challenging but offers exciting research perspectives.

## AUTHOR CONTRIBUTIONS

Josefa Bleu and Marlène Gamelon conceived the study. Bertrand Scaar and volunteers collected the data. Sandrine Zahn developed and performed the sexing measurements. Léo Dejeux curated the data. Josefa Bleu and Marlène Gamelon ran the CMR analyses. Josefa Bleu and Marlène Gamelon wrote the first draft of the manuscript. All authors provided comments on the manuscript and agreed on the final version of the manuscript to be submitted for publication.

## CONFLICT OF INTEREST STATEMENT

The authors declare no conflicts of interest.

## Supporting information


Appendix S1.

## Data Availability

Data (Bleu, 2025) are available on Zenodo at https://doi.org/10.5281/zenodo.15268586.
