# Supplementary material for: Positive covariation between current reproduction and subsequent performance in a raptor: Is the devil in the details?
Source: Ecology. 2025 Jun 2;106(6):e70132. doi: 10.1002/ecy.70132 (PMC12130744; doi:10.1002/ecy.70132)

Positive covariation between current reproduction and subsequent performance in a raptor: Is the devil in the details?

Marlène Gamelon, Bertrand Scaar, Léo Dejeux, Sandrine Zahn and Josefa Bleu

Ecological Applications

Appendix S1. Supplemental Tables and Figures.

Table S1. Number of captures of adults per year between February and July. In these row data, all the captures are counted and not the individuals.

2008	2009	2010	2011	2012	2013	2014	2015	2016	2017	2018	2019	2020	2021
12	13	23	35	25	56	74	98	150	130	122	159	211	202

Table S2: Outputs of the GOF tests performed in U-CARE (in rows) for the three datasets (whole dataset including all individuals (all), on the dataset of known age only (age), and on the dataset of known sex only (sex)). Displayed are the statistics of the tests, the p-values and the degrees of freedom.

Test	Brood size				Brood mass			
	All	Age	Sex Group 1	Sex Group 2	All	Age	Sex Group 1	Sex Group 2
Global	122.393	89.546	108.104	13.526	119.741	88.193	94.380	9.006
	0.841	0.949	0.874	0.999	0.686	0.702	0.930	1.000
	139	113	126	33	128	96	116	28

Figure S1: Map of the nest boxes in Haut-Rhin and Bas Rhin (France).

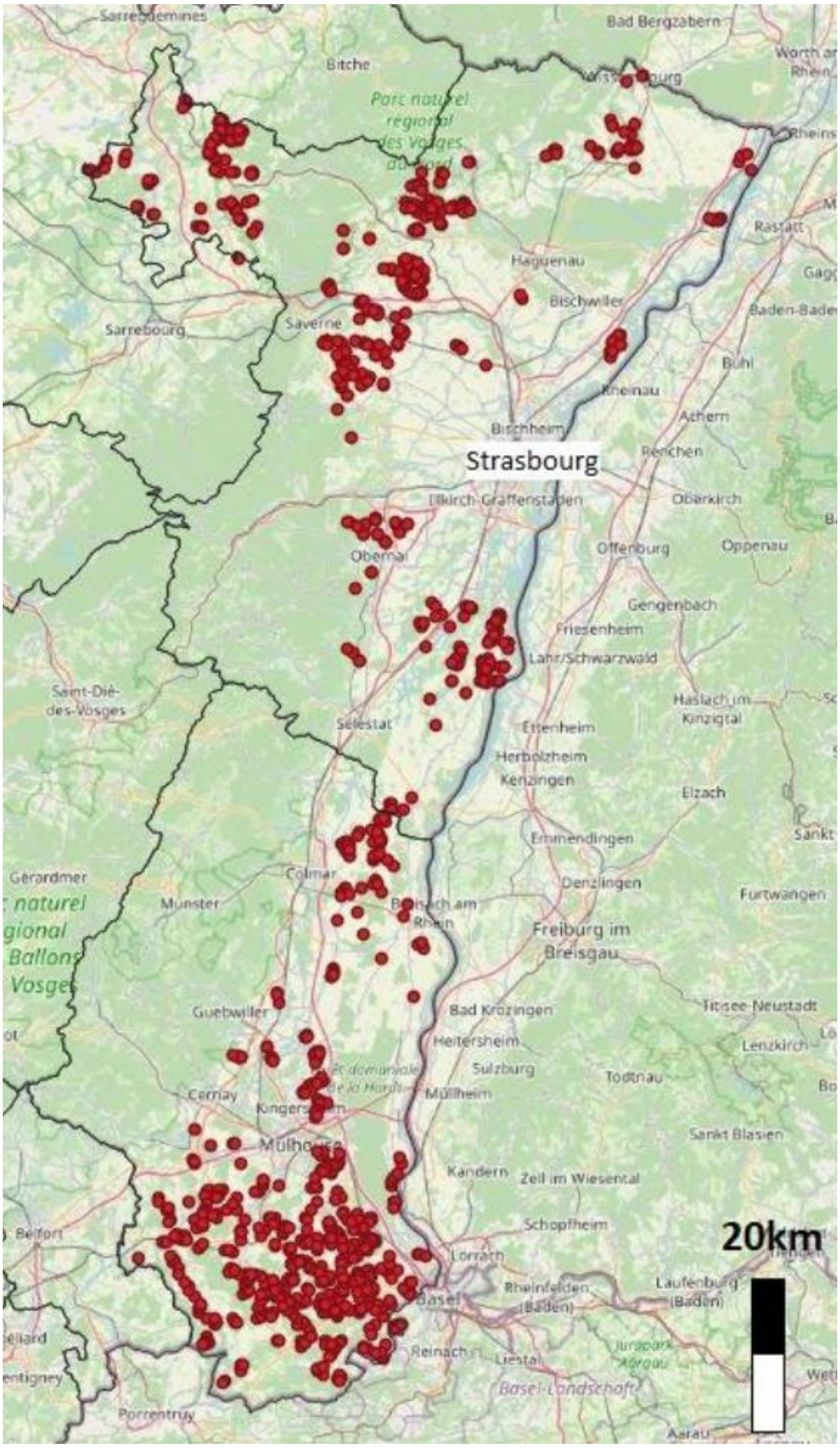

Figure S2: Fate diagram for individuals starting at year *t* with low reproductive success (departure state L) (brood size model). Individuals can survive (S) or die (D) until year *t+1*. For surviving individuals, they can move towards the reproductive state "L" (low reproductive success), "H" (high reproductive success) or remain in the same state "N" (no reproductive success) at year *t+1*. These states can be observed when the individuals are captured (with a probability p_c) and are not observed when the individuals are not captured. States are figured with grey circles (D, N, L, H). The intermediate state S (surviving individuals) is figured with a white circle. Events are figured with squares.

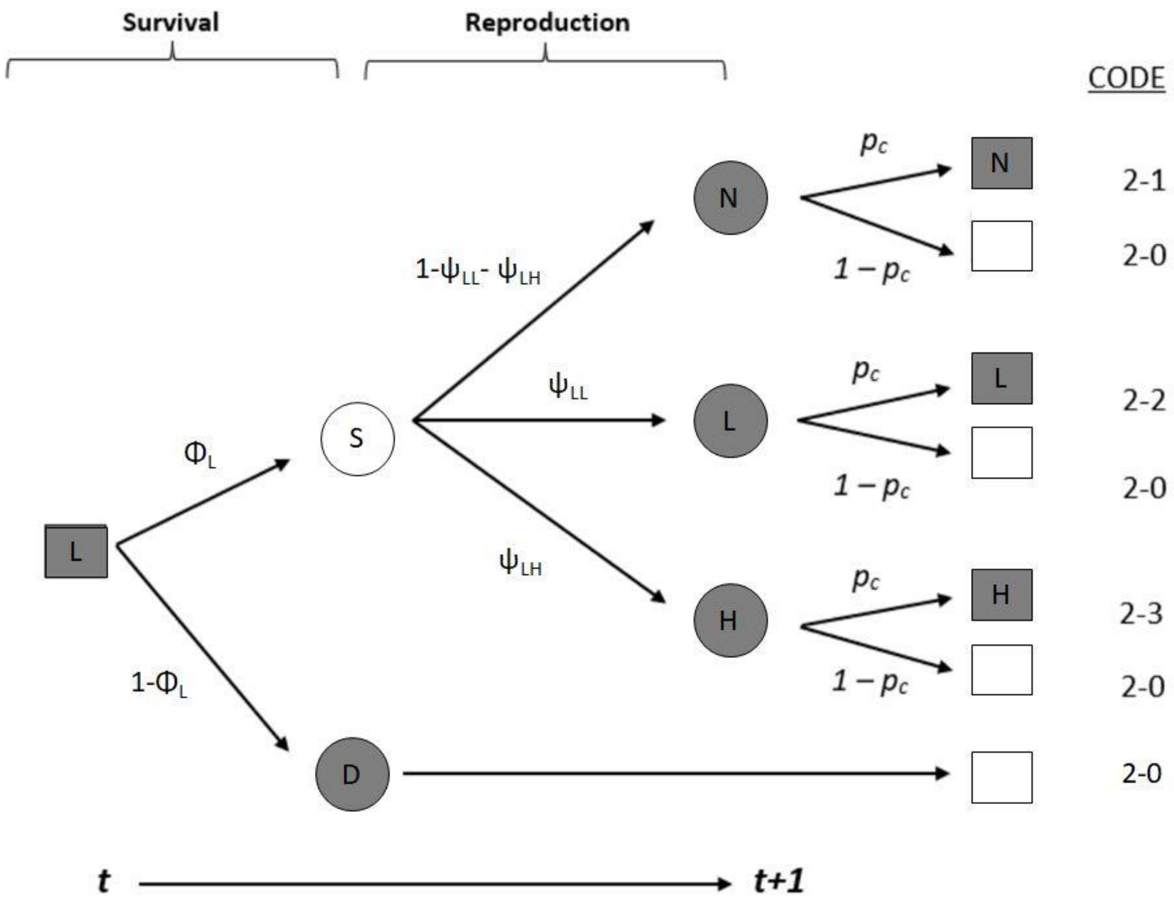

Figure S3: Fate diagram for individuals starting at year *t* with high reproductive success (departure state H) (brood size model). Individuals can survive (S) or die (D) until year *t+1*. For surviving individuals, they can move towards the reproductive state "L" (low reproductive success), "H" (high reproductive success) or remain in the same state "N" (no reproductive success) at year *t+1*. These states can be observed when the individuals are captured (with a probability p_c) and are not observed when the individuals are not captured. States are figured with grey circles (D, N, L, H). The intermediate state S (surviving individuals) is figured with a white circle. Events are figured with squares.

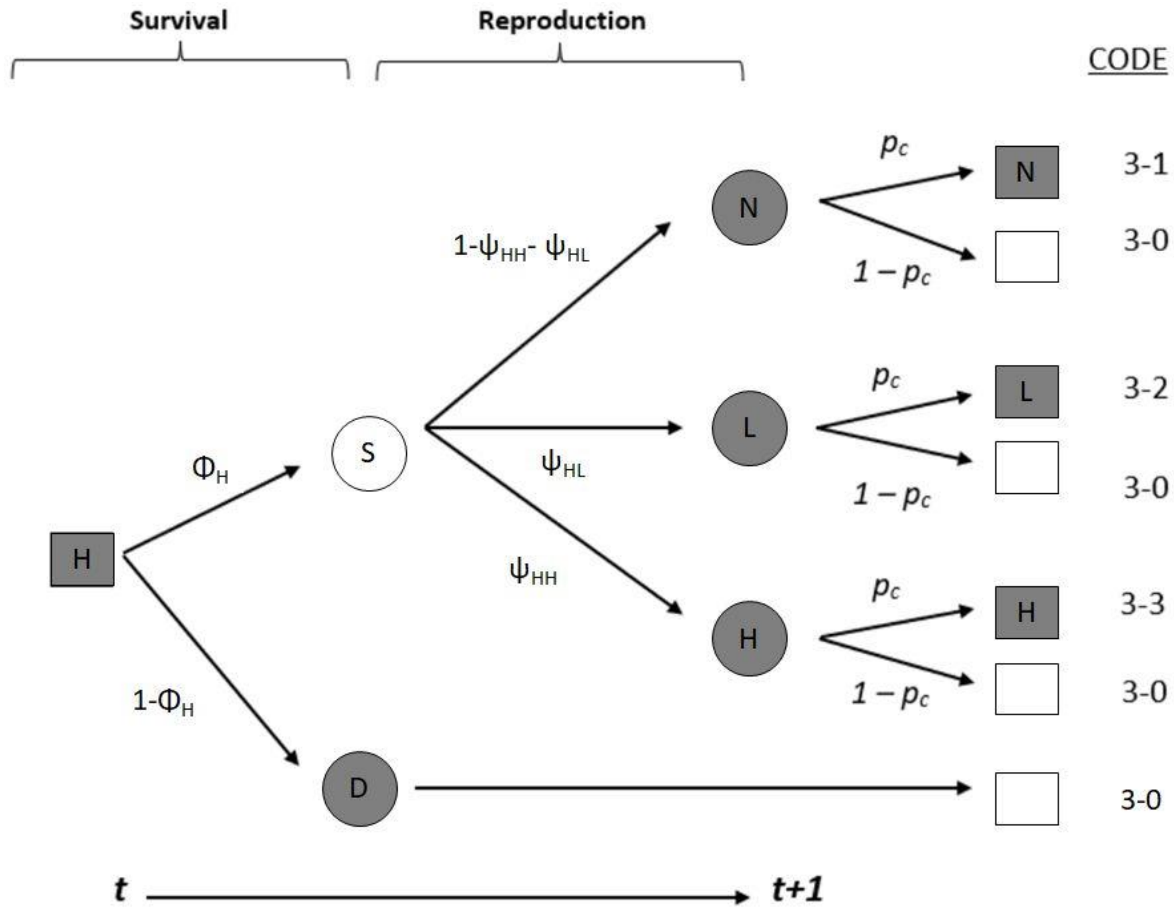

Figure S4: Initial state, transition and event matrixes showing survival probability Φ, breeding probability ψ and recapture probability p_c in brood size analysis. The event matrix for the first capture (B1) is set to one as the first capture has a probability of one.

States:

Γ = {alive non-successful breeder, alive low breeder, alive high breeder, dead} = {N, L, H, D}

Events:

Ω = {non-captured, captured with no reproductive success, captured with low reproductive success, captured with high reproductive success} = {0, 1, 2, 3}

Initial state:

$$\Pi = \begin{pmatrix} \pi_N & \pi_L & 1 - \pi_N - \pi_L & 0 \end{pmatrix}$$

Transition:

$$\Phi = \begin{pmatrix} \varphi_N & 0 & 0 & 1-\varphi_N \\ 0 & \varphi_L & 0 & 1-\varphi_L \\ 0 & 0 & \varphi_H & 1-\varphi_H \\ 0 & 0 & 0 & 1 \end{pmatrix} * \begin{pmatrix} 1-\psi_{NL}-\psi_{NH} & \psi_{NL} & \psi_{NH} & 0 \\ 1-\psi_{LL}-\psi_{LH} & \psi_{LL} & \psi_{LH} & 0 \\ 1-\psi_{HL}-\psi_{HH} & \psi_{HL} & \psi_{HH} & 0 \\ 0 & 0 & 0 & 1 \end{pmatrix}$$

Event:

$$B_1 = \begin{pmatrix} 0 & 1 & 0 & 0 \\ 0 & 0 & 1 & 0 \\ 0 & 0 & 0 & 1 \\ 1 & 0 & 0 & 0 \end{pmatrix}$$

$$B = \begin{pmatrix} 1-p_c & p_c & 0 & 0 \\ 1-p_c & 0 & p_c & 0 \\ 1-p_c & 0 & 0 & p_c \\ 1 & 0 & 0 & 0 \end{pmatrix}$$

Figure S5: Fate diagram for individuals starting at year t with no reproductive success (departure state N) (brood mass model). Individuals can survive (S) or die (D) until year $t+1$. For surviving individuals, they can move towards the reproductive state “L” (low reproductive success), “H” (high reproductive success) or remain in the same state “N” (no reproductive success) at year $t+1$. These states may be observed when the individuals are captured (with a probability p_c) and are not observed when the individuals are not captured. For captured individuals, the brood mass is known with a probability γ_b. States are figured with grey circles (D, N, L, H). The intermediate state S (surviving individuals) is figured with a white circle. Events are figured with rectangles.

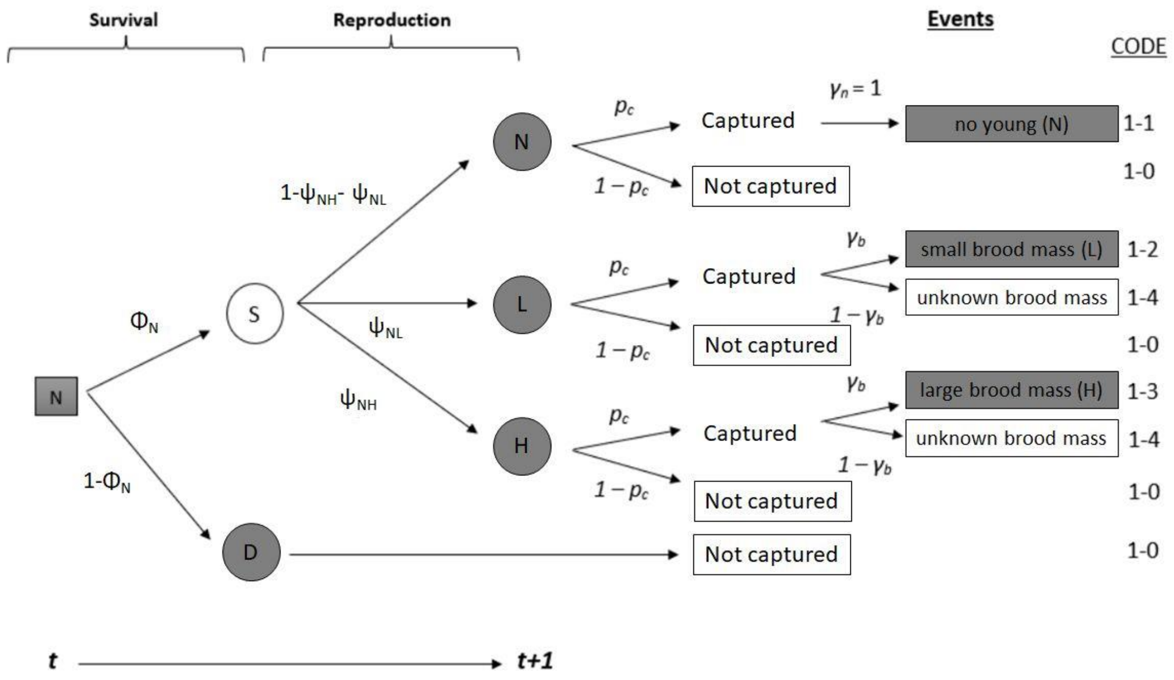

Figure S6: Fate diagram for individuals starting at year *t* with low reproductive success (departure state L) (brood mass model). Individuals can survive (S) or die (D) until year *t+1*. For surviving individuals, they can move towards the reproductive state “L” (low reproductive success), “H” (high reproductive success) or remain in the same state “N” (no reproductive success) at year *t+1*. These states may be observed when the individuals are captured (with a probability p_c) and are not observed when the individuals are not captured. For captured individuals, the brood mass is known with a probability γ_b. States are figured with grey circles (D, N, L, H). The intermediate state S (surviving individuals) is figured with a white circle. Events are figured with rectangles.

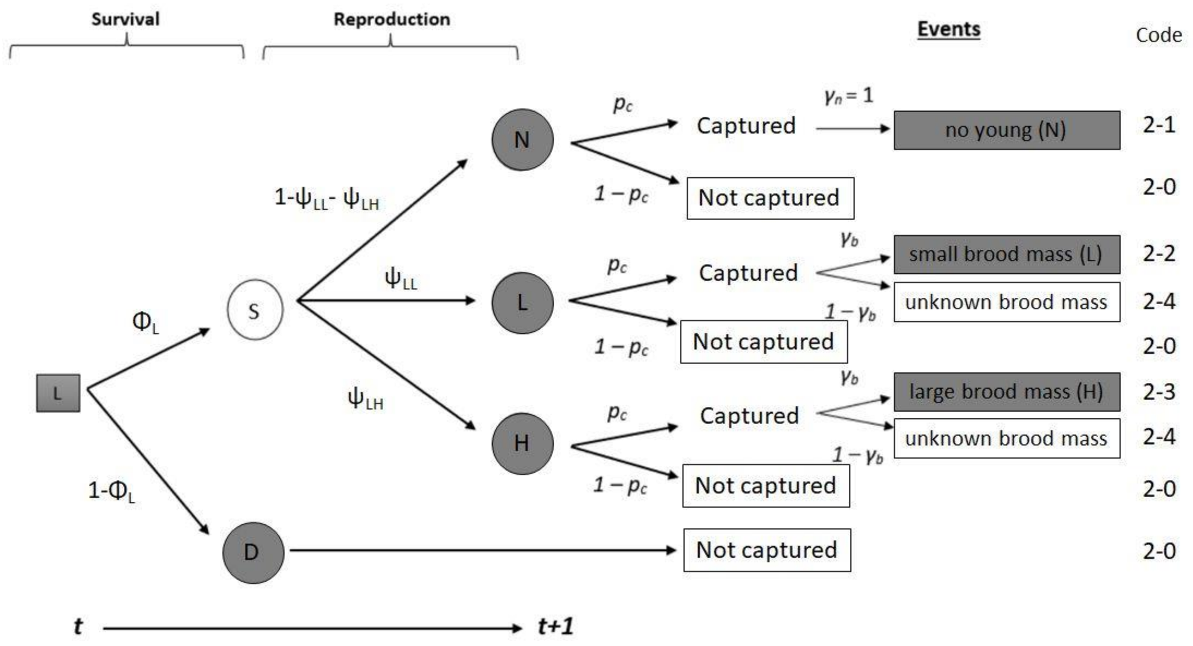

Figure S7: Fate diagram for individuals starting at year *t* with high reproductive success (departure state H) (brood mass model). Individuals can survive (S) or die (D) until year *t+1*. For surviving individuals, they can move towards the reproductive state “L” (low reproductive success), “H” (high reproductive success) or remain in the same state “N” (no reproductive success) at year *t+1*. These states may be observed when the individuals are captured (with a probability p_c) and are not observed when the individuals are not captured. For captured individuals, the brood mass is known with a probability γ_b. States are figured with grey circles (D, N, L, H). The intermediate state S (surviving individuals) is figured with a white circle. Events are figured with rectangles.

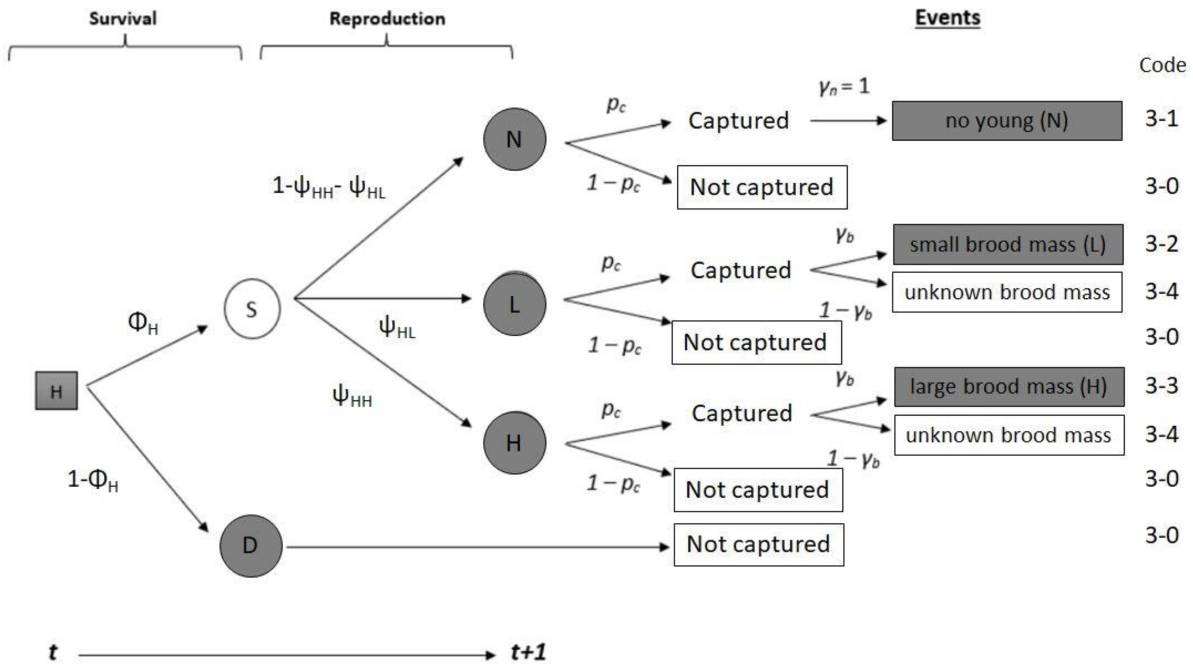

Figure S8: Initial state, transition and event matrixes showing survival probability Φ, breeding probability ψ, recapture probability p_c and the probability of being captured with a known brood mass γ_b in brood mass analysis. The event matrix for the first capture (B1) is set to one as the first capture has a probability of one.

States:

Γ = {alive non-successful breeder, alive low breeder, alive high breeder, dead} = {N, L, H, D}

Events:

Ω = {non-captured, captured with no reproductive success, captured with low reproductive success, captured with high reproductive success, captured with unknown reproductive success} = {0, 1, 2, 3,4}

Initial state:

$\Pi = (\pi_N \quad \pi_L \quad 1-\pi_N-\pi_L \quad 0)$

Transition:

$$\Phi = \begin{pmatrix} \varphi_N & 0 & 0 & 1-\varphi_N \\ 0 & \varphi_L & 0 & 1-\varphi_L \\ 0 & 0 & \varphi_H & 1-\varphi_H \\ 0 & 0 & 0 & 1 \end{pmatrix} * \begin{pmatrix} 1-\psi_{NL}-\psi_{NH} & \psi_{NL} & \psi_{NH} & 0 \\ 1-\psi_{LL}-\psi_{LH} & \psi_{LL} & \psi_{LH} & 0 \\ 1-\psi_{HL}-\psi_{HH} & \psi_{HL} & \psi_{HH} & 0 \\ 0 & 0 & 0 & 1 \end{pmatrix}$$

Event:

$$B_1 = \begin{pmatrix} 0 & 1 & 0 & 0 \\ 0 & 0 & 1 & 0 \\ 0 & 0 & 0 & 1 \\ 1 & 0 & 0 & 0 \end{pmatrix} * \begin{pmatrix} 1 & 0 & 0 & 0 & 0 \\ 0 & 1 & 0 & 0 & 0 \\ 0 & 0 & \gamma_B & 0 & 1-\gamma_B \\ 0 & 0 & 0 & \gamma_B & 1-\gamma_B \end{pmatrix}$$

$$B = \begin{pmatrix} 1-p_c & p_c & 0 & 0 \\ 1-p_c & 0 & p_c & 0 \\ 1-p_c & 0 & 0 & p_c \\ 1 & 0 & 0 & 0 \end{pmatrix} * \begin{pmatrix} 1 & 0 & 0 & 0 & 0 \\ 0 & 1 & 0 & 0 & 0 \\ 0 & 0 & \gamma_B & 0 & 1-\gamma_B \\ 0 & 0 & 0 & \gamma_B & 1-\gamma_B \end{pmatrix}$$

Figure S9: Survival probability between year *t* and *t+1* according to the reproductive state (brood mass) at year *t* when the analysis is performed on the whole dataset (a), on individuals of known age only (b) and on individuals of known sex only (c). Displayed are the estimates provided by the best model retained (see Table 3). Reproductive state at year *t* corresponds to no (N), low (L) or high (H) reproductive success.

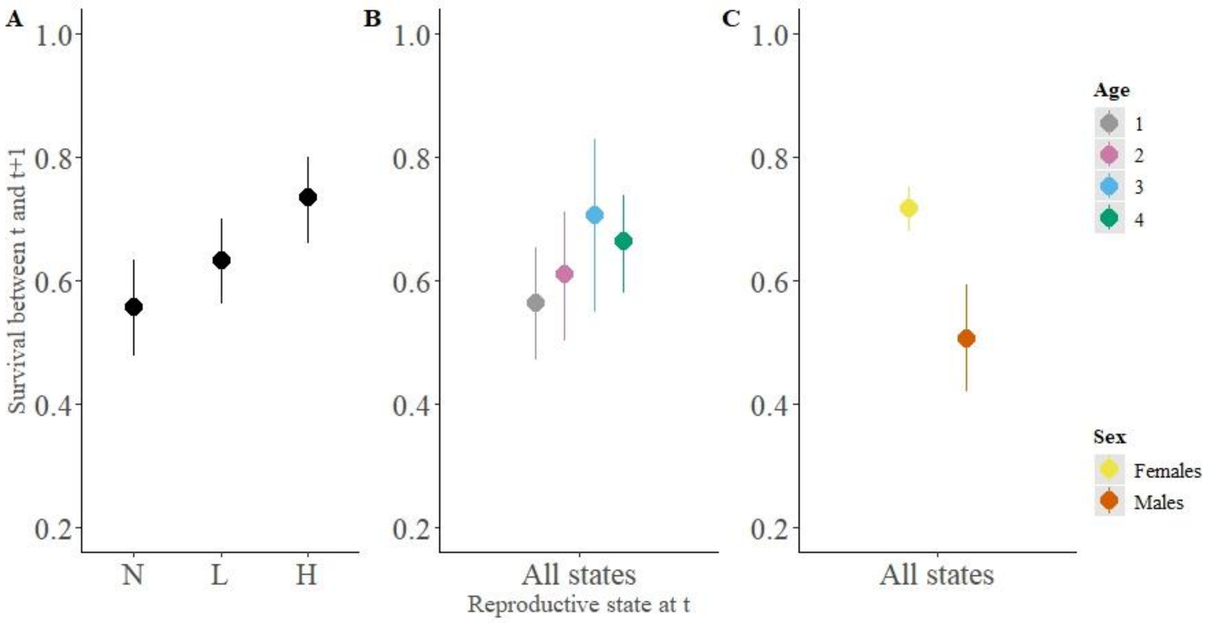

Figure S10: Probability of successful reproduction at year *t+1* according to the reproductive state (brood mass) at year *t* when the analysis is performed on the whole dataset (a), on individuals of known age only (b) and on individuals of known sex only (c). Displayed are the estimates provided by the best model retained (see Table 3). Reproductive state corresponds to no (N) reproductive success and successful breeders (SB).

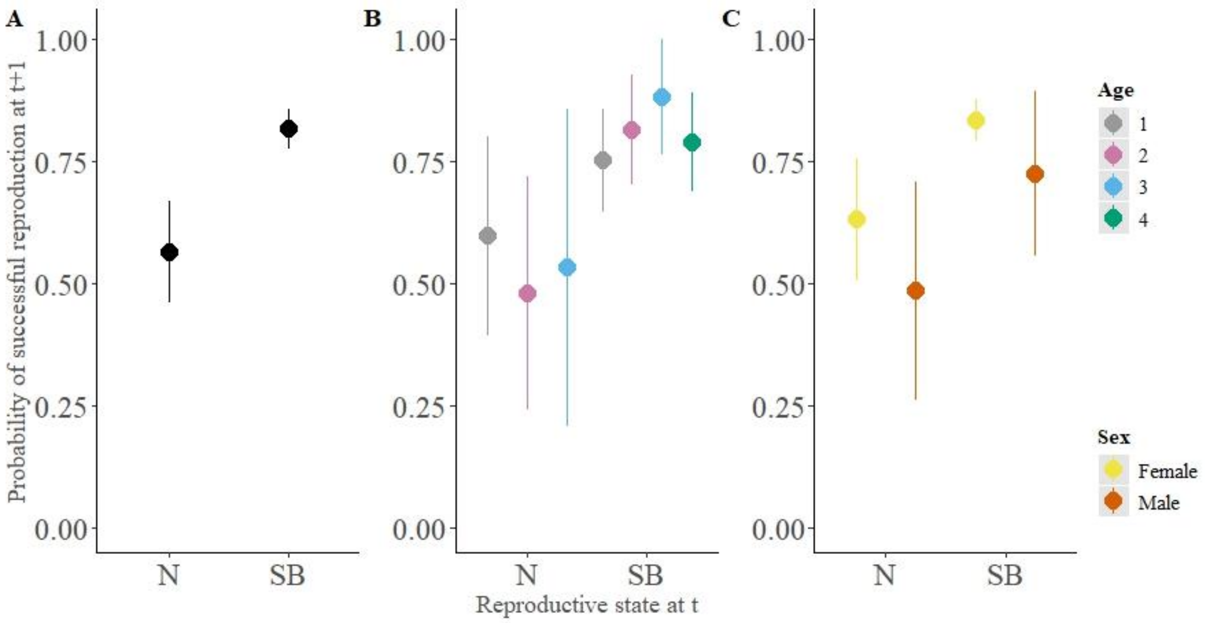

Supplement: Supplementary file 1 — Appendix S1. [file ECY-106-e70132-s001.pdf]
